# A model-based approach to designing developmental toxicology experiments using sea urchin embryos

**DOI:** 10.1007/s00204-021-03201-1

**Published:** 2022-01-13

**Authors:** Michael D. Collins, Elvis Han Cui, Seung Won Hyun, Weng Kee Wong

**Affiliations:** 1 Department of Environment Health Sciences and Molecular Toxicology Interdepartmental Program, University of California, Los Angeles, Los Angeles, CA 90095 USA; 2 Department of Biostatistics, University of California, Los Angeles, Los Angeles, CA 90095 USA; 3 Research Biostatistics, Johnson and Johnson Medical Devices, Irvine, CA 92618 USA

**Keywords:** Approximate design, *D*-optimality, Dose–response, Optimal experimental design, Sea urchin embryo, Trimethoprim

## Abstract

The key aim of this paper is to suggest a more quantitative approach to designing a dose–response experiment, and more specifically, a concentration–response experiment. The work proposes a departure from the traditional experimental design to determine a dose–response relationship in a developmental toxicology study. It is proposed that a model-based approach to determine a dose–response relationship can provide the most accurate statistical inference for the underlying parameters of interest, which may be estimating one or more model parameters or pre-specified functions of the model parameters, such as lethal dose, at maximal efficiency. When the design criterion or criteria can be determined at the onset, there are demonstrated efficiency gains using a more carefully selected model-based optimal design as opposed to an ad-hoc empirical design. As an illustration, a model-based approach was theoretically used to construct efficient designs for inference in a developmental toxicity study of sea urchin embryos exposed to trimethoprim. This study compares and contrasts the results obtained using model-based optimal designs versus an ad-hoc empirical design.

## Introduction

A fundamental concept in toxicology is the dose–response relationship. An initial step in understanding the biological effects of a chemical compound on any organism is the establishment of a dose–response relationship to provide doses of the compound that can be used in subsequent toxicological assessments. Then, after performing the toxicological studies, an understanding of the dose–response relationship allows for extrapolation of the information to predict threshold concentrations, make high- to low-dose predictions and to begin to understand the nature of the interactions of chemical substances in complex mixtures. Although toxicologists frequently use empirical approaches to determine the various dose parameters (e.g., dose levels, spacing, and number of observations per dose) used to experimentally determine a dose–response curve, there are more systematic mathematical approaches of determining the appropriate doses that can yield a reduction in the variance of the relationship. In this era of systems biology where there is an emphasis on providing comprehensive systematic analyses of biological parameters, perhaps, a more systematic approach for establishing dose–response relationships may be warranted. In particular, Giles (2006) noted that only a small percentage of results from laboratory studies can be replicated in clinical trials mainly due to poor design and further lamented that poor designs are responsible for lack of confidence in results. Some possible reasons mentioned included (i) an improper (or lack of a) randomization scheme, (ii) inadequate controls to minimize bias, (iii) inappropriate models, and (iv) a less than thoughtful design strategy and data analysis plan. Study design is therefore a critical aspect of obtaining useful information from toxicology experiments. In particular, a study design can increase the precision in the statistical inference substantially when the choices for the doses are carefully considered. It is therefore suggested that the design of such experiments can likely benefit from a more formal approach to designing experiments where doses can be selected more objectively; to this end, a model-based approach that requires a pre-specified statistical model, a pre-selected design criterion, and a pre-determined design space (dose range) can be helpful. Optimization tools are then used to find designs that optimize the design criterion among all designs on the design space (dose range).

More specifically, a statistical model for the dose–response relationship must be carefully selected upfront to capture all prior information available for the study to formalize the process. This usually requires modeling of how the outcome is related to the dose levels, along with distributional assumptions of the observation errors. The latter are errors incurred in recording the outcome and are beyond the complete control of the research. Standardly, errors are assumed to be independent, identically and normally distributed for inference purposes. There are unknown parameters in the model, and usually, some are more meaningful than others. The functional relationship between the mean outcome and the doses, which may also include other explanatory variables in the regression model, is usually user-selected based on the experience of the investigator or from the literature. The model is linear if this relationship depends on the parameters in a linear fashion; otherwise, it is a nonlinear model. Examples of linear models are polynomial models or fractional polynomial models and examples of nonlinear models are exponential models with the simple one or two parameters or the Michaelis–Menten model and its several variations.

At the onset of the study, the real goal or goals of the experiment must be formulated in terms of a design or optimality criterion as accurately as possible. Optimality needs to be clearly defined, even for studies where it may be difficult to do so. The design criterion enables one to compare competing designs using a mathematical approach to facilitate decisions on how to select or improve the experiment for maximal gain in statistical efficiency with minimal resources.

In a dose–response experiment, decisions regarding the dose range, the number of doses, the dose levels, and the number of experimental units at each dose are sometimes made predicated on nebulous criteria. These are design issues that can potentially have a substantial impact on the quality of the statistical inference at the end of the study, yet they are decided in some cases on an ad-hoc basis. Frequently, an equal number of experimental units are assigned at each dose. When the doses are equally spaced, these are called uniform designs in the statistical literature and while they are appealing and intuitive, it has been shown that they can be inefficient, depending on the goal of the study and the underlying model assumed. For example, Wong and Lachenbruch (1996) showed that performance of such designs can depend sensitively on the choice of the number of doses in a uniform design, the model, and the optimality criteria. Therefore, each aspect in the design of the study must be carefully considered to realize maximum accuracy in the information. Such attention to detail will enhance reproducibility, thus addressing a current issue in animal experimentation (Giles 2006).

To optimally design an experiment, model assumptions are required to work out the mathematical and statistical details. Invariably, the goal is formulated as an objective function defined on the user-specified dose range (or design interval) that depends on the statistical model and the design. The optimization of the criterion can then be performed among a specific class of designs, for example, among all designs with five doses, or among all designs on a given dose interval. The resulting optimal design is therefore model-based and, as a consequence, can be highly model-dependent, suggesting that choice of a statistical model for the dose–response study is also important. Wong (1994) is one of many design papers in the statistical literature that provides examples that showed efficiencies of a design can vary substantially when model assumptions are violated.

The approach used in this study was to utilize data from an empirically designed study to essentially model developmental toxicity dose–response relationships from the purple sea urchin, *Strongylocentrotus purpuratus*, after early embryo exposure to the antibiotic trimethoprim. Trimethoprim has been dichotomously categorized in the human as either a teratogen or as a non-teratogen depending on the assessor. If it is a human teratogen, it has been described as idiosyncratic but weakly teratogenic (Shepard et al. 2002). For decades, human teratogens have been used to induce dysmorphogenesis in developing sea urchins with the goal of studying the basic biology of the chemical perturbation (Hagström and Lönning 1973; Estus and Blumer 1989a, b; Sconzo et al. 1996; Qiao et al. 2003; Buznikov et al. 2007; Reichard-Brown et al. 2009). These studies have been predicated on the idea that the developmental cell processes and functions of sea urchins and humans may share common chemical perturbations despite the difference in organs between the species (e.g., sea urchins lack liver, kidney, lung, and brain). A rationale for using sea urchin embryos in this study is that replicate sample populations are relatively simple and inexpensive to produce, allowing for experiments with large numbers of fertilized eggs. It is hypothesized that humans and sea urchin share many homologous genes (Venter et al. 2001; Sea Urchin Sequencing Consortium 2006). It is further hypothesized that the chemical target of a teratogenic substance can be determined by perturbing the sea urchin developmental gene regulatory network (GRN) which is a highly experimentally derived gene regulatory network (Martik et al. 2016). Essentially, two developmental outcomes were modeled in this study, namely the developmental abnormality aboral radialization and developmental arrest/death. Aboral radialization is a dysmorphic form of a sea urchin embryo/pluteus in which the left–right axis has been lost to become radially symmetrical and instead of having oral and aboral ectoderm (the sea urchin equivalent of dorsal–ventral); all ectoderm is aboral. The goal is to demonstrate how modeling and optimal design can contribute to the process of producing dose–response curves in this specific experiment, with the idea that these concepts are more generalizable in the establishment of dose–response relationships for a variety of toxicological analyses.

## Materials and methods

### Sea urchin developmental toxicity tests

Experiments consisted of culturing purple sea urchin (*Strongylocentrotus purpuratus*) fertilized eggs through the first 96 h post-fertilization (hpf). For brevity, data for two endpoints of interest are reported at 96 hpf after exposure from 1 to 24 hpf to various doses of trimethoprim, namely (1) early developmental arrest or death (EDA/D) of the zygote, and (2) a developmental abnormality called aboral radialization.

Sea urchins were originally captured off the coast of San Diego, California. They were initially transferred to Kerckhoff Marine Laboratory at Corona del Mar in California and subsequently delivered to the Davidson/Peter Laboratory at the California Institute of Technology, Pasadena, CA. They were maintained in aquaria, containing sea water taken directly from the Pacific Ocean (Kerckhoff Marine Laboratory), that are temperature controlled to 16 ± 1 °C. The tanks were aspirated with air and supplied with kelp. These tanks were maintained in a room with a temperature of 16 ± 2 °C. Sea urchins were either manually shaken or injected with 0.55 M potassium chloride to induce gamete release. Sperm were collected in 20–100 microliter aliquots in 0.5 ml Eppendorf tubes and were stored at 4 °C for up to 7 days. Eggs were collected on the day of fertilization and rinsed three times with Millipore-filtered sea water (MPFSW). Then, sperm were mixed with MPFSW (1:2–1:5 dilution) and 1 or 2 drops of the solution were placed in a beaker with the rinsed eggs. The time when the sperm are placed in the vessel with the eggs is presumed to be the time of fertilization and subsequent steps were monitored in terms of hours post-fertilization (hpf). Approximately 90 s after the sperm were placed with the eggs, a small volume of eggs (300–500) were visualized under a dissecting microscope to detect the fertilization membrane. Fertilization rates were usually greater than 98%. If the eggs had an appropriate fertilization rate (> 95%), then the number of fertilized eggs in the culture was counted by diluting the eggs (1:30) in MPFSW and counting eight 200 µl aliquots in a glass micropipette. After determining the concentration of fertilized eggs, the eggs were diluted to a concentration of approximately 1500 eggs per ml. The eggs were then placed in a glass 125 ml flask with a volume of 75 ml of MPFSW at a density of 20 eggs per ml. The flasks were then placed on a platform shaker at a rate of 120 rpm for the duration of the experiment. At 1 hpf, trimethoprim (100 mM stock solution in dimethyl sulfoxide) was placed in some flasks containing the sea urchin embryos at various concentrations and a control flask was administered a quantity of dimethyl sulfoxide (DMSO) equivalent to the largest volume of the stock solution used in the experiment. Each 125 ml flask containing approximately 1500 embryos had a specific concentration of trimethoprim (ranging from 0 to 1000 µM). A number of flasks were cultured simultaneously to produce a concentration–response curve. The MPFSW containing the various concentrations of trimethoprim were exchanged for MPFSW without trimethoprim at 24 hpf, so that the duration of exposure was approximately 23 h (although the compound within the embryo is not removed by this process). The sea urchin embryos were then maintained under the culture conditions until 96 hpf, a time at which the organisms should develop to the pluteus stage. At approximately 96 hpf, the cultures are sampled and about 100 individual plutei more or less from each flask were examined under a dissecting microscope to determine the phenotype. The morphological characteristics of each of the examined plutei were recorded.

The biological endpoints evaluated in this study for the purpose of modeling were aboral radialization (Radial: Ab) and early developmental arrest and/or death (EDA/D). The compound was placed in a flask with developing sea urchin embryos at 1 h post-fertilization (hpf), the sea water containing the compound is replaced at 24 hpf, and then, the embryos are assessed at 96 hpf. At the time of phenotyping the plutei (96 hpf), some or all of the organisms may not have advanced to the pluteus stage. In other words, the developing sea urchin embryos may have stopped developing at an earlier stage in development. When the embryo has developed at 96 hpf to a stage achieved by a normal sea urchin embryo at 24 hpf (gastrulation) or less, it is categorized as developmentally arrested and/or dead. On observation, it is difficult to distinguish if the embryos are developmentally arrested (but viable) or dead (non-viable). If a chemical is sufficiently water soluble, then at some dose, it will eventually produce this endpoint and, as a result, almost all compounds that have been examined in the sea urchin embryo assay produce this phenotype at high doses. Depending on the concentration of the compound, the embryos may stop development at an early developmental stage (e.g., 2 hpf) or at a later developmental stage (e.g., 23 hpf), with higher chemical concentrations inducing earlier developmental arrest/death. These two categories of arrest/death phenotypes, namely early or late, were not delineated in this study.

Aboral radialization is a relatively specific endpoint that is only produced by a relatively small number of compounds studied to date. Radialization was originally characterized by Hardin et al. (1992) where nickel chloride induced oral radialization, but aboral radialization was reported by Bergeron et al. (2011) following exposure of sea urchins to either chlorate or the TGF beta type 1 receptor inhibitor SB-431542. The difference between these two phenotypes is that the larvae are either surrounded by epithelia with red pigment cells (aboral) or not (oral). For the purposes of modeling, both of these endpoints are binary.

### Statistical methods for modeling the sea urchin concentration–response relationship

The Beta model is a relatively simple model for studying a continuous outcome with values confined to between 0 and 1. Ordinarily, it has a single controlled variable that fluctuates across a pre-specified range of doses called the design space. Experiments are conducted using selected doses from the design space and determining the outcome. One potential experimental outcome is the percentage of embryos with developmental delay or death at a particular dose; this observed outcome varies between 0 and 100 %, or 0 and 1 after standardization.

A most basic statistical model to study proportions or an outcome that takes values between 0 and 1 is the Beta model with two parameters and a single controlled variable. The controlled variable is the chemical concentration in our experiments. A log-logistic model with additive normal errors could also be used to study a continuous outcome between 0 and 1. The model is relatively simple to operationalize and interpret, since it has only two parameters. The four-parameter log-logistic model (Ritz 2010) is a more flexible model in that it can also fit outcomes when their values are not restricted to between 0 and 1. It is also a widely used model with readily available software for data fitting and analysis in dose–response studies. For example, Ritz, et al. (2015) and Knezevic, et al. (2021) provide a dose–response analysis using the R software for the four-parameter log-logistic model. The Beta model has the same mean response as the four-parameter log-logistic model when the latter is assumed to have values of the responses that are bounded by 0 and 1. The Beta model is increasingly used to fit proportions and rates using a single variable across disciplines and is well discussed in various textbooks in statistics. An example of using such a model and one of its extensions to estimate the parameters in the model is Ferrari and Cribari-Neto (2004). For instance, one of its extensions is a re-parameterization of the Beta model, so that its two parameters are the mean and dispersion parameters to study the regression model and examine departures from the model assumptions. While estimation issues are quite well studied for this and other statistical models, design issues for different models are less investigated, even though it is clear that how data are collected can seriously affect the quality of the inference to follow.

Before collecting data in an experiment, design issues should be considered and addressed as fully as possible. They include making decisions on how many dose levels to be included in the study, what concentrations or doses, and how many experimental units to assign at each dose. The latter are called replicates where there are repeated observations taken under the same experimental setting. These questions are difficult or impossible to answer objectively without a statistical model in mind. Typically, previous studies on similar agents or related analogues can provide some information on the dose–response relationship for the chemical agent under investigation. The choices for the number of doses, their concentrations, and the replication schemes to be used in the study are the design components, and they can affect the quality of the statistical inference. Careful choices of these variables can provide information as to whether the assumed statistical model is adequate by conducting a lack of fit test commonly described in design and analysis monographs; see, for example, Montgomery (2012).

The typical estimates of interest in a toxicology experiment are the model parameters, or functions thereof, which may include the various quantiles typically used for estimating various lethal or risk assessment thresholds. For example, one may suspect hormesis occurs for a compound with a curvilinear mean response curve and there is interest to estimate the dose where the turning point in the dose–response curve occurs. If the expected curve has a quadratic form, i.e., Ey = *a*_0_ + *a*_1_*x* + *a*_2_*x*^2^, where x is the dose level and a_0_, a_1_, and a_2_ are the coefficients in the linear model, then taking the derivative of Ey with respect to x and setting it equal to zero yields the turning point, which is x*= − *a*_1_/(2*a*_2_). In this case, the model is clearly linear and the function of interest to estimate is a nonlinear function of the model parameters. This means that the asymptotic variance of the estimated x* contains model parameters and a locally optimal design cannot be implemented unless prior estimates of the model parameters are available. Consequently, design issues can become complex quickly even for a relatively simple linear model. Likewise, when a logistic model is used to study a binary response variable y, for instance, it may be desirable to estimate a lethal dose or concentration (LC_p_), which can be expressed as a nonlinear function of the model parameters. For instance, to estimate the LC_5_, the concentration expected to produce a 5% death rate in embryos, a commonly used statistical technique called the Delta’s method is first applied to obtain the (asymptotic) variance of the estimated LC_5_ before the variance is minimized by choice of the design. The design problem is more complex, because the design criterion also contains the unknown parameters. Again, this means that nominal values are required before a locally optimal design can be implemented. Alternatively, in both examples, more complicated design strategies like adopting a Bayesian approach or a maxim in design strategy can also be used. The former assumes that a prior distribution for the unknown parameters is available to average out the unknown parameters before the criterion is optimized. In the latter case, it is required that there is a known set of plausible values for the true parameters and one seeks a design to maximize the minimum efficiency arising from choices of the model parameter in the plausible set.

How does one use statistical ideas to design experiments with quality inference when a statistical model seems plausible to begin with? The model-based design approach begins with calculating the likelihood function from the error distribution assumed in the model. In statistical estimation, a key object to focus on is the Fisher Information matrix defined by the expected value of the second derivatives of the log-likelihood function with respect to the model parameters. This matrix depends on model parameters when the model is nonlinear and it does not when the model is linear. This explains why designing for a nonlinear model is generally more difficult than for a linear model.

The design criterion is a scalar function of the information matrix, and frequently, it is formulated, so that it has some desirable properties. For example, it is commonly formulated as a concave or convex function of the information matrix, and so, a design that maximizes the design criterion is sought. For example, the D-optimality criterion for estimating model parameters is formulated as the log of the determinant of the information matrix and a design that maximizes the criterion is a D-optimal design. Because such a design minimizes the generalized variance of the model estimates, a D-optimal design provides the smallest confidence ellipsoids for the model parameters. If there is only one parameter in the model, this means that a D-optimal design provides the shortest confidence interval for a given confidence level. Likewise, the design criterion c-optimality is used to find a design that is best for estimating an interesting function of the model parameters. A c-optimal design minimizes the (asymptotic) variance of the estimated function and such an optimal design produces the most accurate estimate of the function of interest. As mentioned earlier, these design criteria typically depend on the unknown model parameters and cannot be directly optimized.

The simplest way to overcome the issue is to find locally optimal designs. This requires prior estimates (sometimes called nominal values) of the unknown parameters by a knowledgeable party or information from previous studies on the compound or similar compounds. These nominal values are then substituted as values for the unknown model parameters, so that optimization can proceed as described. The resulting optimal design clearly depends on the nominal values and they are called locally optimal. There are some similarities with the ad-hoc empirical approach that is generally used by toxicologists, except that they do not assume any statistical model and do not attempt to have the most accurate statistical inference for the given experimental cost.

Locally D-optimal designs for estimating parameters in the Beta model were reported in Latif and Yab (2015). An extension of locally optimal designs is to assume that there are various competing nominal values from previous studies and experts. This situation can arise when different prior studies or experts offer differing information of the anticipated dose–response curve. Instead of relying on a single best guess for the parameters, a prior density will have to be elicited from the competing information to come up with a prior density to describe the plausible values of the true model parameters. Bayesian D-optimal designs are designs that maximize the D-optimality criterion after the design criterion has the unknown parameters averaged over the prior density. Some Bayesian optimal designs for the Beta model were reported for a regression setup in Jafari and Pirmohamdi (2017). Wu et al. (2005) used the Beta model to model responses from a drug trial and showed the D-optimal design performed well for the application. This means that the optimal experimental design produces estimates for the model parameters that are most accurate among other designs given the same setup.

**Fig. 1:**
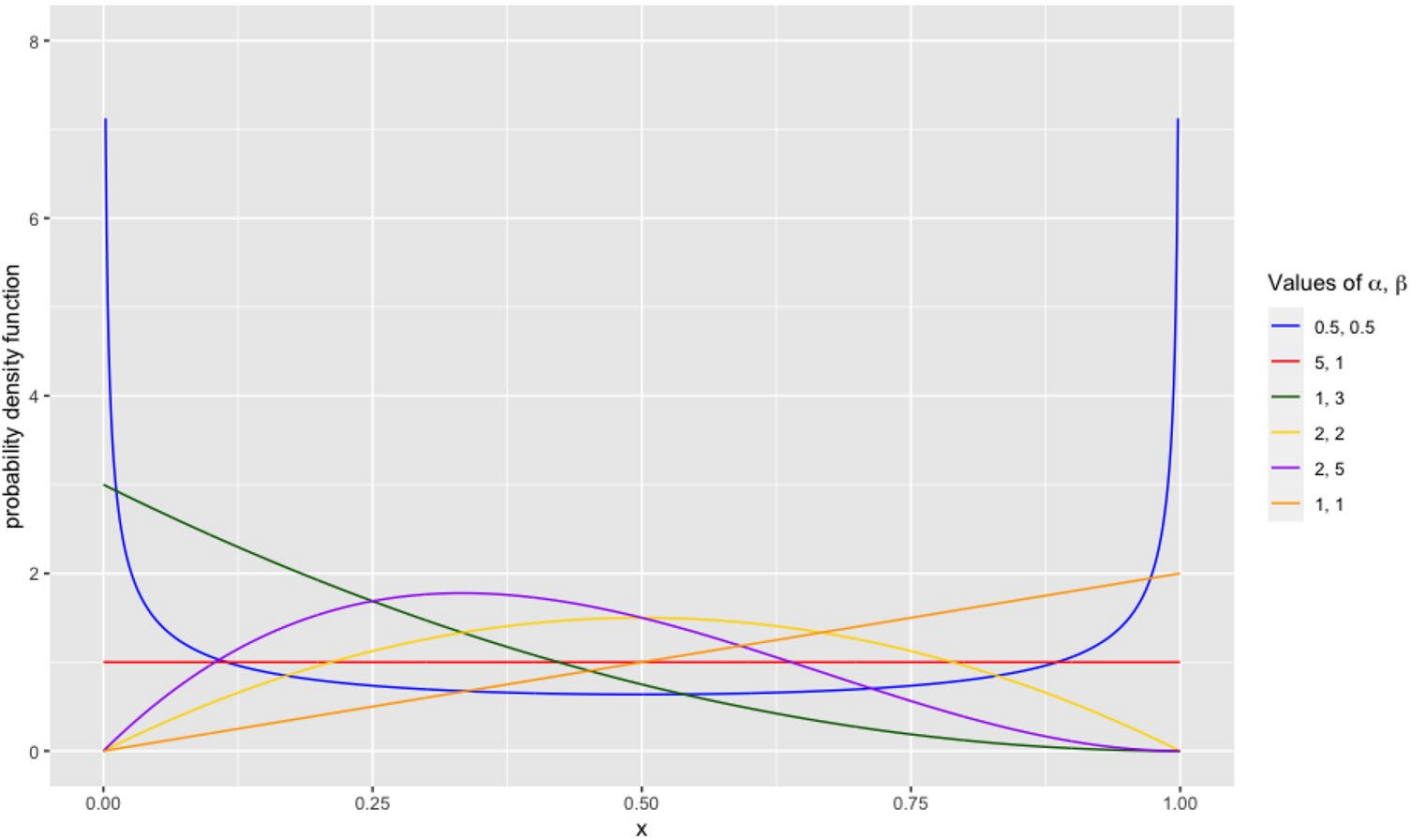
Density of beta distribution with parameters α and β

The density of the response rate *y* under the Beta model is given by

$$f(y) = \frac{{\Gamma \left( {\alpha + \beta } \right)}}{\Gamma \left( \alpha \right)\Gamma \left( \beta \right)}y^{\alpha - 1} (1 - y)^{\beta - 1} ,$$

where 0 ≤ *y* ≤ 1 and Γ is the Gamma function. Figure 1 shows its shapes for various values of the two parameters α and β.

Wu et al. (2005) showed that the parameters in the Gamma function can be parametrized in terms of the dose by letting $a = \exp (\alpha_{1} + \alpha_{2} x)$ and $\beta = \exp (\beta_{1} + \beta_{2} x)$, so that under such a setup, the mean response rate at dose level *x* is

$$\frac{1}{{1 + \exp \{ (\beta_{1} - \alpha_{1} ) + (\beta_{2} - \alpha_{2} )x\} }},$$

where Θ = (*α*_1_, *α*_2_, *β*_1_, *β*_2_) is a set of unknown model parameters that controls the shape of the response curve and the dose level x is the actual dose range (no need to standardize between 0 and 1 or transfer to log scale). The Beta model describes a sigmoidal response curve and it can be shown that there are inhomogeneous variances for the response rates y across the dose levels.

Optimal designs can also be constructed for estimating percentiles in a distribution. For instance, if there is interest in estimating the concentration for which it will result in a user-specified percentage, say p, of the sea urchins experiencing death, i.e., the LC_p_, one can find a design that minimizes the (asymptotic) variance of the estimate for LC_p_. This variance can be calculated based on the model. Alternatively, a dual-objective optimal design may be sought primarily for estimating a specific LC_p,_ and second, to estimate the model parameters as accurately as possible. Table 3 displays some of these theory-based designs.

## Results

### Sea urchin concentration–response data

**Fig. 2:**
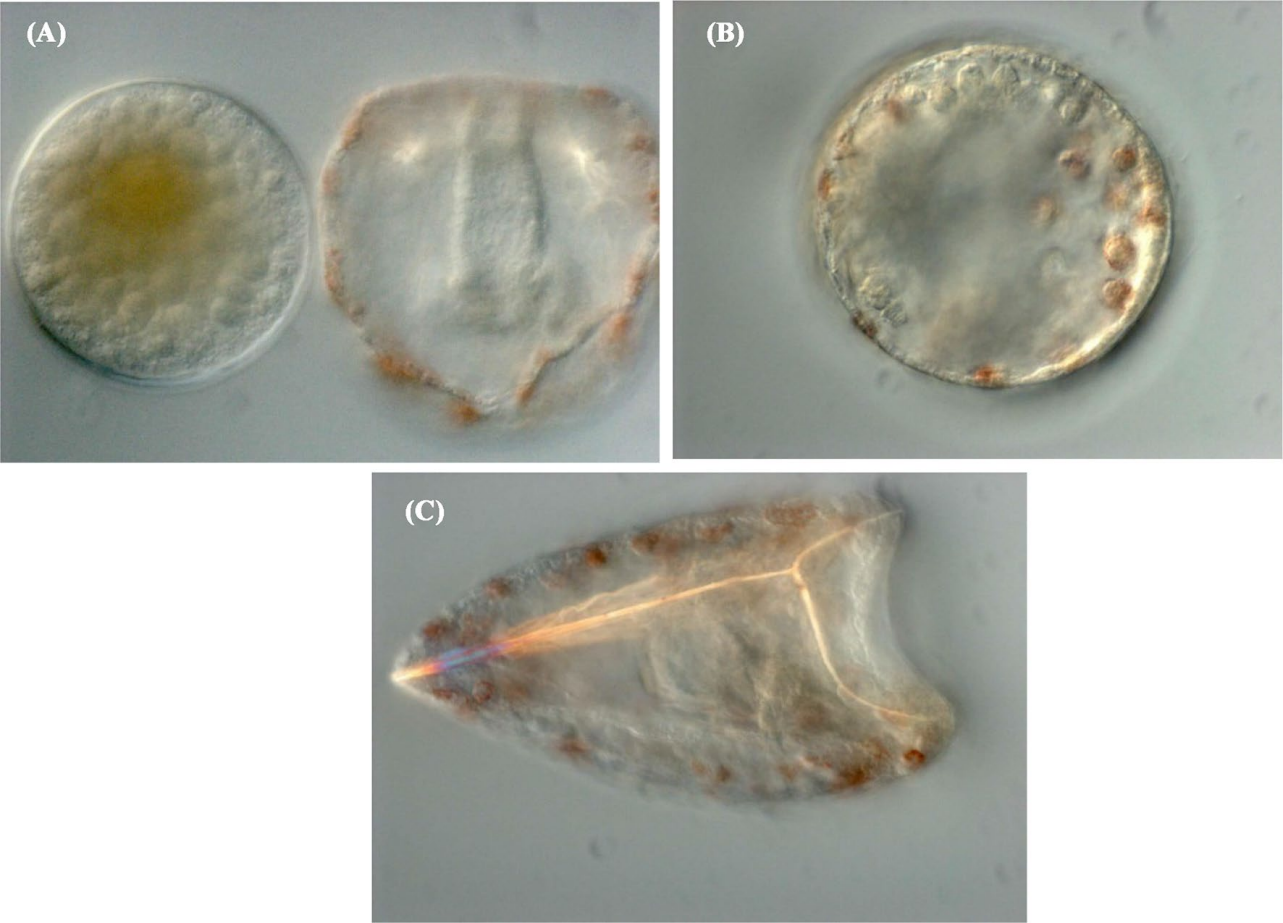
Development of sea urchin embryos. **A** Shows two sea urchin embryos/plutei that were exposed to 100 µM trimethoprim from 1 to 24 hpf and observed at 66 hpf. The pre-pluteus on the left has the phenotype described as early embryonic developmental arrest/death (EDA/D), and the pluteus on the right is phenotypically normal for this time of development. **B** This embryo was exposed to 180 µM trimethoprim from 1 to 24 hpf and observed at 72 hpf and has aboral radialization that is difficult to observe, because there is a problem getting the spicules in focus simultaneously due to the depth of field issues. Nevertheless, there are spicules located at approximately positions of the clock of 8, 9, 10, 11, 1, 3, and 4. **C** The pluteus shown is a control that was given vehicle (DMSO) from 1 to 24 hpf and then observed at 72 hpf. This photograph shows a single skeletal spicule in the foreground and a second spicule that is less focused but meets the first spicule on the aboral (left) region of the embryo

Figure 2 shows photomicrographs of sea urchin larvae at either 66 or 72 hpf that have either been exposed to trimethoprim from 1 to 24 hpf or exposed to vehicle (DMSO). This figure illustrates embryos/larvae with the two phenotypes that have been described in this study, namely early embryonic developmental arrest/death (panel A: left side) and aboral radialization (panel B), along with two larvae that have normal phenotypes for the observation time (panel A: right side and panel C). Table 1 shows numerical phenotypic data for sea urchin embryos that were exposed to various concentrations of trimethoprim from 1 to 24 hpf and then observed at 96 hpf for the purpose of deriving concentration–response information. The columns in the table include the dose of trimethoprim, the total number of embryos/plutei examined (N), number of normal plutei (normal), number of embryos with early embryonic developmental arrest/death (EDA/D), and aboral radialization (Radial: Ab). The response rate at each dose level for EDA/D was computed by dividing the number of arrested/dead embryos by the number of observations. The response level for aboral radialization was derived by dividing the number of larvae with aboral radialization by *N*, the number of presumed living plutei (total number of examined plutei/embryos minus EDA/D) (Fig. 2).

**Table 1:** Concentration–response data for the sea urchin study with various trimethoprim concentrations (Conc) and results for the two endpoints: (1) embryonic developmental arrest/death (EDA/D), and (2) aboral radialization (Radial:Ab).

Conc (uM)	*N*	Normal	EDA/D	Radial: Ab	Conc (uM)	*N*	Normal	EDA/D	Radial: Ab
0	93	87	0	0	200	113	0	113	0
1000	83	0	83	0	0	91	89	0	0
0	97	93	2	0	100	105	17	2	0
100	94	56	10	0	150	108	0	3	25
300	87	0	78	0	175	103	0	37	59
1000	95	0	95	0	200	108	0	60	48
0	87	84	1	0	0	89	88	0	0
100	93	59	1	0	100	121	50	0	0
300	80	0	80	0	125	107	6	0	1
1000	70	0	70	0	150	118	0	5	35
0	94	87	7	0	175	115	0	1	35
100	89	55	11	0	200	114	0	4	54
200	104	0	20	65	0	105	102	2	0
300	129	0	129	0	150	104	9	0	2
0	128	104	17	0	300	107	0	77	28
100	106	61	7	0	0	109	104	1	0
150	111	15	1	33	150	100	1	34	25
200	112	0	51	34	225	104	0	40	25
300	75	0	75	0	300	104	0	104	0
0	88	88	0	0	450	98	0	98	0
100	104	80	2	0	0	101	100	0	0
150	106	45	2	2	150	107	0	1	11
200	101	0	4	33	225	101	0	21	63
300	97	0	27	48	300	100	0	100	0
0	89	81	1	0	450	100	0	100	0
100	97	75	2	4	0	101	97	97	0
150	120	0	59	48	150	102	6	2	89
200	93	0	30	62	225	101	0	34	67
300	69	0	69	0	300	102	0	80	22
0	90	88	1	0	450	100	0	97	3
100	105	96	1	0	0	109	108	1	0
150	103	74	2	2	150	116	35	5	10
200	124	16	9	60	0	107	107	0	0
300	111	4	47	53	180	113	3	0	96
0	100	94	2	0	0	120	120	0	0
100	103	93	1	0	180	101	0	0	98
200	106	0	80	4	0	103	101	1	0
300	114	0	112	0	180	64	0	51	13
0	100	99	0	0					
100	103	25	11	3					
150	97	0	88	7					
200	88	0	88	0					
300	106	0	106	0					
0	100	92	3	0					
100	107	81	1	0					
150	109	0	20	17					

*N* is the total number of embryos examined, and normal is the number of embryos that developed in the normal range

### Modeling and optimal designs of the concentration–response data

For any set of data, there are several statistical models that may fit the data well. There are various measures in the statistical literature that assess how well a particular model fits the data. Some of these measures are information-based criterion or are based on how well the fitted model predicts the response. Various models were compared using goodness of fit measures, such as the pseudo R^2^, the deviance, the likelihood value, and the Akaike’s Information Criterion. The Beta model was found to have a significantly better fit than other models for the sea urchin data and it is used in this study.

The algorithm used to find single and dual-objective locally optimal designs for the Beta model was written in R and similar to the ones in Hyun et al. (2018) and Hyun et al. (2020). This algorithm was chosen, because it is based on a state-of-the-art exchange algorithm proposed by Yang, et al. (2013) and comes with a proof of mathematical convergence. For convenience, this algorithm is named the YBT algorithm after the surnames of the three coauthors of the paper.

The ad-hoc empirical design implemented in the sea urchin study was compared with the various optimal designs found by the YBT algorithm using the same dose ranges. Using data from an earlier experiment, the assumed nominal parameters for the Beta model were the maximum likelihood estimates (MLEs), which were Θ_1a_ = (− 1.250, 0.004, 1.460, − 0.007) for the first endpoint and Θ_1b_ = (1.513, − 0.013, 6.103*,* − 0.034) for the second endpoint.

Where there is no previous information regarding a compound of interest, a common approach would be to administer log doses such as 1, 10, 100, and 1000 to establish limits and then use more linear doses once the limits of the dose–response curve are determined. In the current study, the concentration levels between the extremes were used to ascertain the slope of the response curve. For the first endpoint, arrest rates (i.e., EDA/D/N), the implemented design is designated as *ξ*_*o*1_. From Table 1, it can be seen that it has 11 different concentration levels in the range *X* = [0, 1000] and replications at various concentrations have unequal numbers of sea urchin embryos.

For the second endpoint, aboral radialization rates (i.e., Radial:Ab/(*N*-EDA/D)), the implemented design is designated by *ξ*_*o*2_ and is the same as *ξ*_*o*1_ except that it has the restricted concentration range *X* = [0, 450]. The design space was selected, because the aboral radialization endpoint cannot be observed at concentrations higher than 450 µM due to the death or arrest of all the embryos at these levels.

Both implemented designs for the sea urchin study were empirical and this is a common approach for studies conducted in various laboratories. The experimental design in this circumstance was based on literature information on the biological activity of trimethoprim, the experience of the investigator, as well as results from earlier experiments to influence decisions in later experiments. Results from the experiments showed that the proportions of both endpoints respond as a sigmoidal response curve with inhomogeneous variances as the level of trimethoprim varies.

Are the above results accurate and reliable? Would another investigator examining the same endpoints using sea urchins come up with similar results? From the practical standpoint, there may be a long list of possible causes for why a scientist would not be able to duplicate the results of an experiment whether they were in the same or a different laboratory and the list would not only include the various underlying models that were used to address this issue or any of the mathematical manipulations that are being considered. Therefore, the quality of the statistical inference at the end of the study must be addressed for others to have faith in the toxicological findings. Giles (2006), mentioned earlier, alluded to such issues in toxicology experiments and traced them to a root cause, which is the use of inefficient designs in such studies. How is it possible to judge the experimental results and what comparisons should be made with the results to assess quality?

Implementing more efficient designs provides an answer, and since optimal designs are not without drawbacks, as noted before, additional design strategies can be developed to address practical problems. The next section discusses the situation where there is uncertainty about the nominal values for the model parameters and a desire to assess the relative robustness of the locally optimal design to misspecification in the nominal values.

### Locally optimal designs for the Beta model

**Fig. 3:**
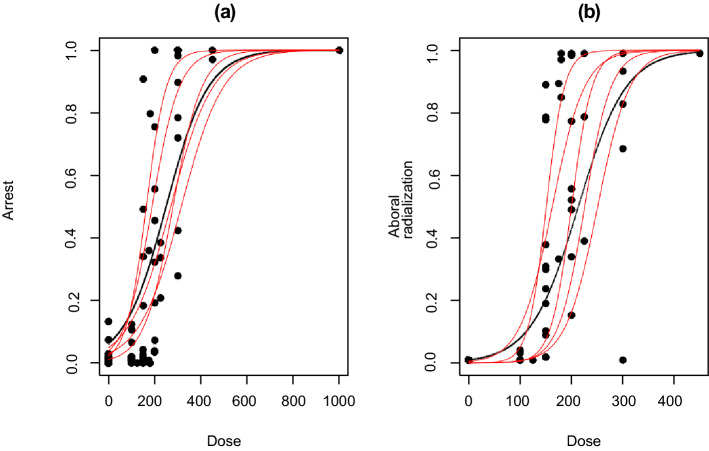
Response curves from the six sets of the parameter values for each endpoint. The black solid curve represents the response curve from the MLEs as nominal values and the red curves are from the five additional parameter sets. **a** Early embryonic developmental arrest/death (Arrest) versus concentration (Dose) relationship. **b** Aboral radialization versus concentration (Dose) relationship

The locally optimal design determined by the algorithm depends on the nominal values of the parameters, and if the distance is far from the true parameter values, the locally optimal design becomes inefficient. Since the true parameters are unknown, it is important to investigate if the locally optimal design is sensitive to the misspecification of the nominal values. To investigate if the optimal designs were robust to misspecified nominal parameter values, five additional sets of parameter values were selected that provide reasonable approximations to response curves for both of the endpoints. Table 2 displays the six selected parameter sets and Fig. 3 shows the fitted response curves for the two endpoints.

**Table 2:** Six selected sets of parameter values that provide reasonable response curves for the sea urchin data

(a) Arrest/death rates	(b) Aboral radialization rates
Θ_1a_ = (− 1.250, 0.004, 1.460, − 0.007)	Θ_1b_ = (1.513, − 0.013, 6.103, − 0.034)
Θ_2a_ = (─1.500, 0.007, 1.700, − 0.010)	Θ_2b_ = (0.900, − 0.021, 6.103, − 0.052)
Θ_3a_ = (− 1.750, 0.010, 2.000, − 0.013)	Θ_3b_ = (0.900, − 0.021, 8.930, − 0.053)
Θ_4a_ = (− 1.500, 0.004, 1.460, − 0.007)	Θ_4b_ = (0.900, − 0.021, 9.700, − 0.060)
Θ_5a_ = (− 1.000, 0.004, 2.460, − 0.007)	Θ_5b_ = (1.100, − 0.015, 9.700, − 0.072)
Θ_6a_ = (− 1.000, 0.006, 3.460, − 0.010)	Θ_6b_ = (1.500, − 0.015, 11.500, − 0.065

The sets of nominal parameter values Θ_1a_ and Θ_1b_ are the MLEs obtained from fitting the data to the first and second endpoints, respectively, and the sets Θ_2–6_ are departures from the MLEs

A locally *D*-optimal design for the Beta model was generated from the algorithm for each of the six sets of nominal values for the model parameters. These designs depend on the nominal values and can perform poorly if the nominal values are misspecified. It is therefore desirable to have a design robust to misspecification in the model parameters. One version of a robust *D*-optimal design is to maximize a weighted average of the *D*-optimality criteria across the six parameter sets with each weight proportional to the probability that the set of nominal values is the true value or close to the true value. Table 2 displays robust *D*-optimal design for the two endpoints under the Beta model where it has been assumed that all the sets of parameter values are equally likely to be valid and they are denoted as *ξ*_*RD*_.

The algorithm in Hyun and Wong (2015) was modified to find designs for estimating model parameters and the concentration *LC*_*p*_ (lethal concentration for percentage p of the population) for which a user-specified *p*% of the urchins would succumb. This is an example of a dual-objective optimal design where there are two pre-determined objectives at the onset. For the sea urchin study, it was assumed both objectives are equally important and the six sets of parameter settings are equally likely to be true or close to the true values of the model parameters.

Table 3 displays robust optimal designs and dual-objective optimal designs for estimating model parameters and one of four lethal concentrations: LC_10_, LC_20_, LC_25_, and LC_50_. The construction strategy for these designs is similar to those for constructing robust D-optimal designs in Hyun and Wong (2015), where more technical details are available. From Table 3, it can be observed that robust designs and dual-objective optimal designs require 3–6 dose levels and all contain the lower and upper limits of the dose range. For example, if the primary goal is to estimate parameters in the Beta model for the first endpoint, the robust *D*-optimal design in the first row of column (a) allocates about 34*.*1% of the embryos to the zero dose level, 3*.*2% of the embryos to the 250 dose level, 37*.*5% of the embryos to the 300 dose level, and 25*.*2% of the embryos to the 1000 dose level.

**Table 3:** Optimal designs for both endpoints under the Beta model: the first row displays the selected dose levels and the second row displays the corresponding weight

Design	(a) Arrest rates	(b) Aboral radialization rates
ξ_RD_	$$\left[ {\begin{array}{*{20}c} 0 & {250} & {300} & {1000} \\ {0.341} & {0.032} & {0.375} & {0.252} \\ \end{array} } \right]$$	$$\left[ {\begin{array}{*{20}c} 0 & {163} & {207} & {450} \\ {0.450} & {0.154} & {0.166} & {0.230} \\ \end{array} } \right]$$
ξ_RDc10_	$$\left[ {\begin{array}{*{20}c} 0 & {236} & {1000} \\ {0.453} & {0.414} & {0.133} \\ \end{array} } \right]$$	$$\left[ {\begin{array}{*{20}c} 0 & {158} & {213} & {450} \\ {0.285} & {0.225} & {0.377} & {0.113} \\ \end{array} } \right]$$
ξ_RDc20_	$$\left[ {\begin{array}{*{20}c} 0 & {186} & {246} & {1000} \\ {0.324} & {0.130} & {0.417} & {0.129} \\ \end{array} } \right]$$	$$\left[ {\begin{array}{*{20}c} 0 & {158} & {212} & {226} & {450} \\ {0.253} & {0.258} & {0.125} & {0.258} & {0.106} \\ \end{array} } \right]$$
ξ_RDc25_	$$\left[ {\begin{array}{*{20}c} 0 & {213} & {264} & {1000} \\ {0.286} & {0.307} & {0.277} & {0.130} \\ \end{array} } \right]$$	$$\left[ {\begin{array}{*{20}c} 0 & {158} & {163} & {212} & {226} & {450} \\ {0.246} & {0.154} & {0.116} & {0.091} & {0.287} & {0.106} \\ \end{array} } \right]$$
ξ_RDc50_	$$\left[ {\begin{array}{*{20}c} 0 & {271} & {298} & {1000} \\ {0.203} & {0.540} & {0.127} & {0.130} \\ \end{array} } \right]$$	$$\left[ {\begin{array}{*{20}c} 0 & {164} & {212} & {226} & {450} \\ {0.233} & {0.289} & {0.034} & {0.335} & {0.109} \\ \end{array} } \right]$$

It is counter-intuitive that the optimal design allocates about a quarter of the observations to the 1000 dose level, which is the highest dose in this study. The high dose was selected in part, because it is believed that at such a dose, 100% of the experimental units will result in death/arrest, and furthermore, it would be anticipated that there would be essentially no variability in this response. Does it make sense that such a large percentage of embryos be allocated to this dose? Perhaps not, but optimal design construction is solely based on mathematical and optimization considerations only and so the optimal design may deviate from the pragmatic concerns of a particular experimental goal. A general rule for the application of optimal design is that it should be used as an overall guide in the design of experimental studies and should incorporate pragmatic study considerations which are used to modify the optimal design. For example, it may be deemed desirable to have a smaller percentage assigned at the highest dose and augment the resulting design with additional doses predicated on particular biological activities. The modified design can then be assessed by calculating its efficiencies under different model assumptions and criterion. The implemented design should have acceptably high efficiencies, say 80% or higher, so that the altered design is a compromise between statistical efficiency and pragmatic consideration. Of course, the term `high’ is a relative term and depends on the investigator and the problem at hand.

In general, designs with high efficiencies are sought. Formally, if *D*-optimality is the design criterion, the *D*-efficiency of a design is defined by the ratio of the determinant of its information matrix to that from the *D*-optimal design. For interpretability, the ratio is raised to *p*th root where *p* is the number of parameters in the model. A design with 80% *D*-efficiency can be interpreted as requiring 1/0.80 = 1.25 times more observations to perform as well as the *D*-optimal design for estimating the model parameters. Thus, a design with the *D*-efficiency close to 1 signifies that the design performs just as well as the *D*-optimal design, and a design with very low *D*-efficiency close to 0 signifies that it performs very poorly for estimating model parameters relative to the *D*-optimal design.

**Fig. 4:**
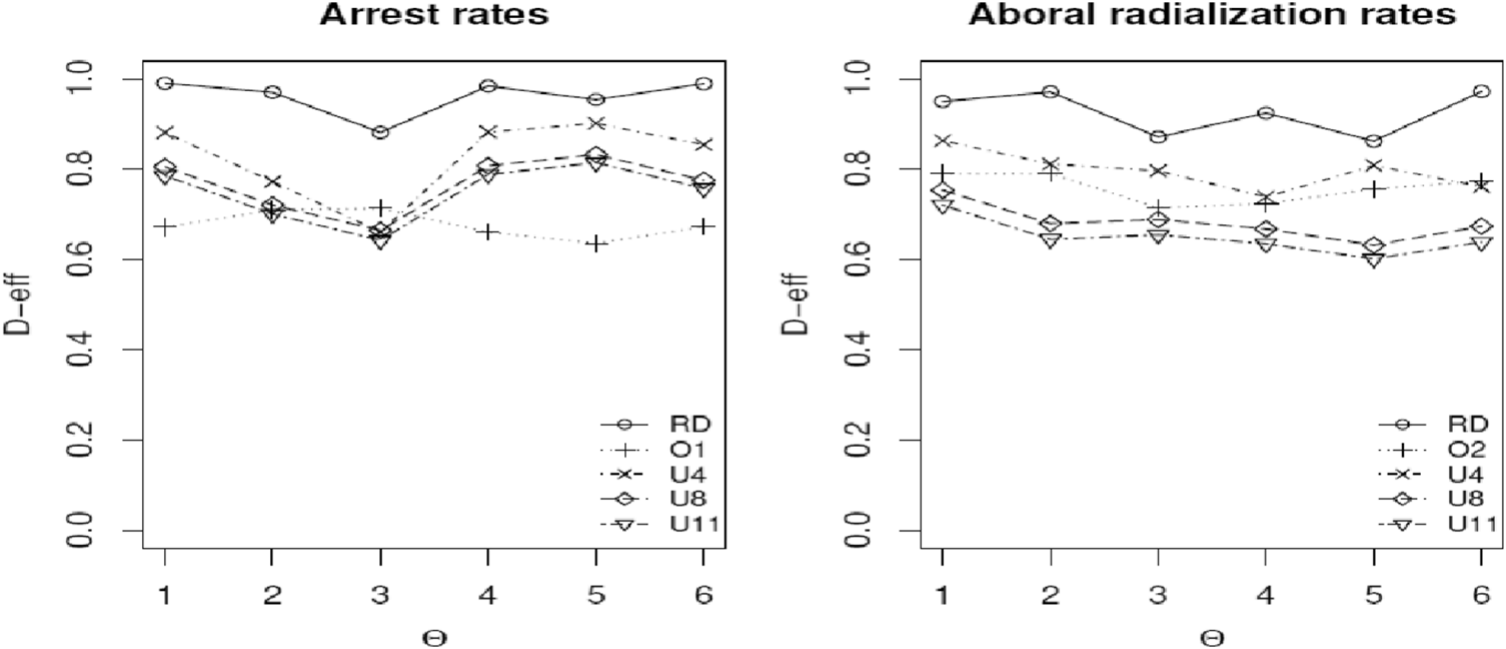
*D*-efficiencies of the various designs relative to the locally *D*-optimal designs with MLEs as nominal values for estimating model parameters across the six sets of nominal parameter values for both endpoints. RD stands for the *ξ*_*RD*_, O1 and O2 stand for ad-hoc empirical implemented designs, *ξ*_*o*1_ and *ξ*_*o*2_, respectively, and U4, U8, and U11 stand for the uniform designs *U*_4_, *U*_8_, and *U*_11_, respectively

**Fig. 5:**
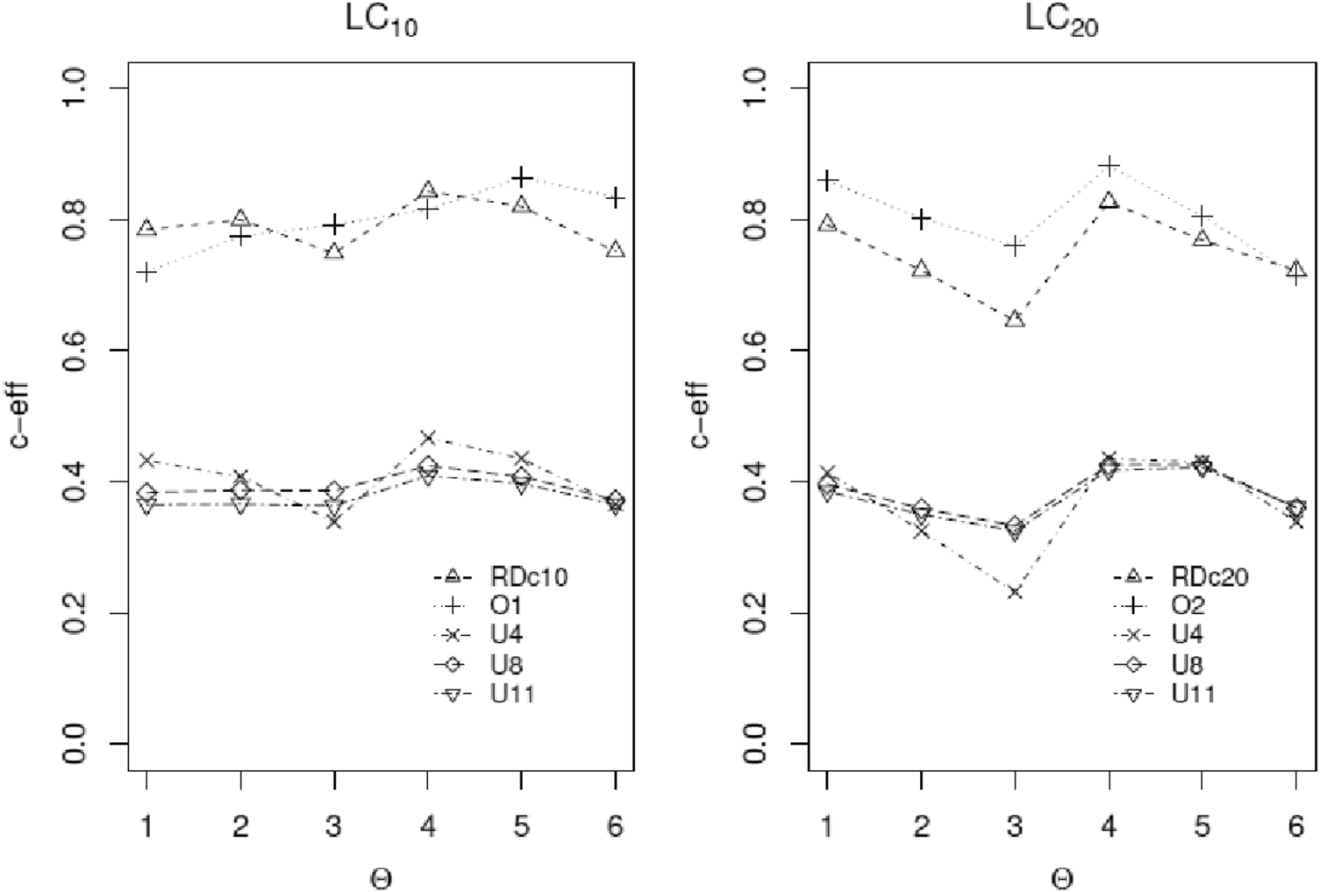
*C*-efficiencies of various designs relative to the locally *c*-optimal designs with MLEs as nominal values for estimating the LC_10_ and LC_20_ across the six sets of nominal parameter values for the arrest/death rates as the first endpoint. RDc_10_ and RDc_20_ stand for the robust optimal designs, O1 stands for the ad-hoc empirical design *ξ*_*o*1_ used for the study, and *U*4,* U*8, and *U*11 are the uniform designs *U*_4_, *U*_8_, and *U*_11_, respectively. The notation O2 stands for the ad-hoc empirical design *ξ*_*o*2_ obtained from O1 but on a restricted design space

**Fig. 6:**
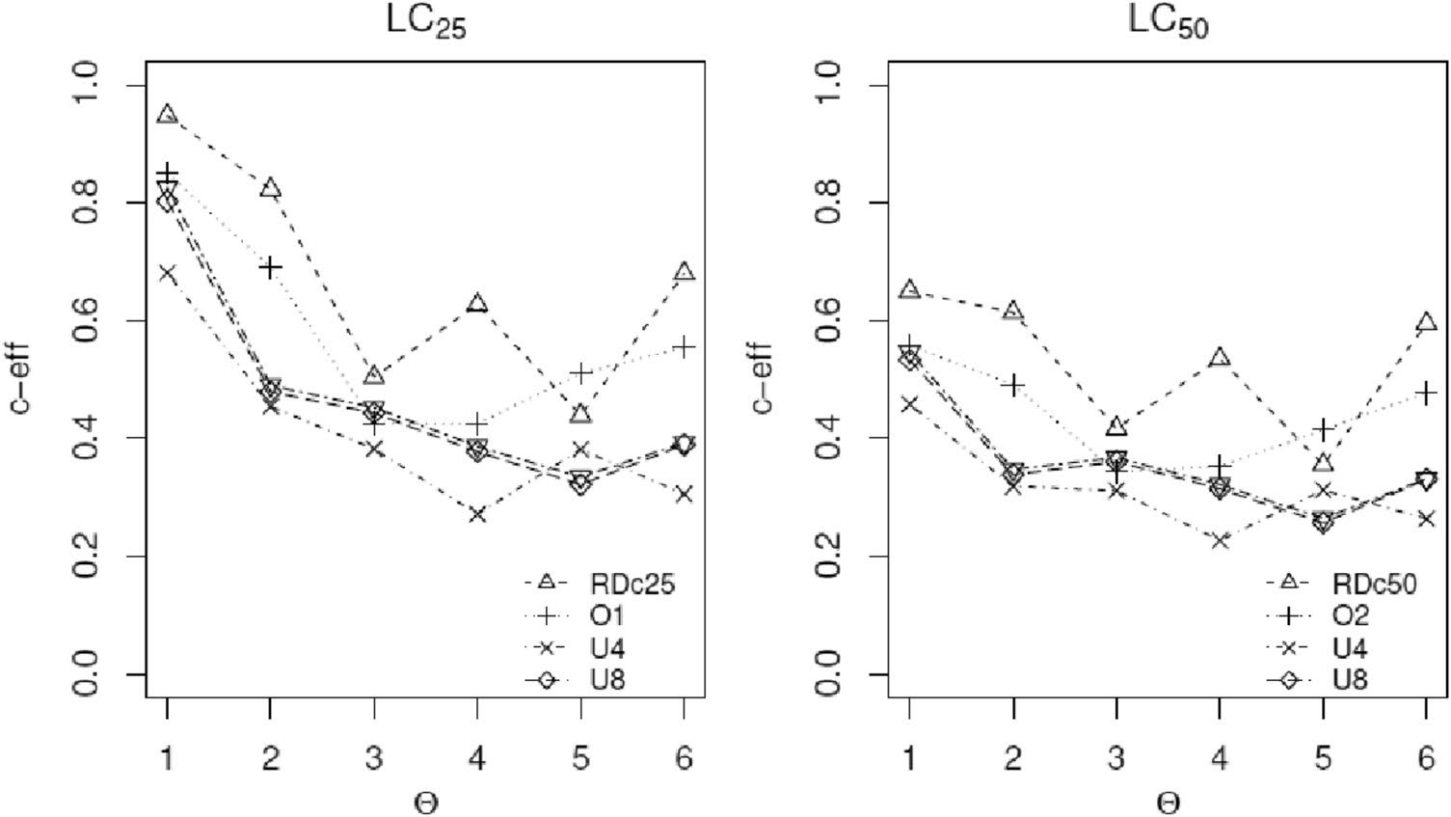
C-efficiencies of various designs relative to the locally *c*-optimal designs with MLEs as nominal values for estimating the LC_25_ and LC_50_ across the six sets of nominal parameter values for aboral radialization as the second endpoint. RDc_25_ and RDc_50_ stand for the robust optimal designs, O2 stands for the ad-hoc empirical design *ξ*_*o*2_ used for the study, and *U*4, *U*8, and *U*11 are the uniform designs *U*_4_, *U*_8_, and *U*_11,_ respectively. The notation O1 stands for the ad-hoc empirical design *ξ*_*o*1_ implemented in the study to obtain the responses over the full dose range of interest

The definition for c-efficiency is the ratio of the variance of the estimated function using the c-optimal design to that from the design of interest. The interpretation of c-efficiency is similar to that for *D*-optimality. Figure 4 displays the *D*-efficiencies and Figures 5 and 6 show c-efficiencies of various designs for estimating LC_10_ and LC_20_, and LC_25_ and LC_50_, respectively, when different sets of nominal parameters are assumed. A design with consistently high efficiencies across different sets of nominal values is always appealing, because there is always uncertainty in the true values of model parameters. The same reasoning holds for wanting to have a design with high efficiencies across different models and criteria.

### Robustness properties of the designs

The robustness properties of the optimal designs *ξ*_RD_ and *ξ*_RDcp_ and the ad-hoc empirical designs (*ξ*_*o*1_ and *ξ*_*o*2_) to the misspecified nominal model parameters are compared for both endpoints. Note that (i) the empirical design *ξ*_*o*1_ is the implemented design on the whole dose range for the first endpoint (arrest rates), and *ξ*_*o*2_ is the same as O1 but on a restricted or truncated dose range for the second endpoint (aboral radialization rates), and (ii) robust designs are simply designs found after averaging the nominal values for the model across the six sets with equal weights. For studying the two endpoints, the focus is on estimating lower lethal concentrations (LC_10_ and LC_20_) for the first endpoint and higher concentrations (LC_25_ and LC_50_) for the second endpoint. The performance of a few uniform designs relative to the optimal designs and the ad-hoc empirical designs were assessed. Uniform designs are designs with equally spaced doses across the dose interval and have equal number of observations at each dose. They are popular due to their simplicity and intuitive appeal. If a uniform design has *k* points, it is denoted by *U*_k_, and the performances of the optimal designs found here are compared with those of *U*_k_ for selected values of *k* = 4, 8, or 11.

Figure 4 is a plot of the *D*-efficiencies of the various designs across different sets of nominal values for the model parameters. It shows the merits of the robust *D*-optimal design *ξ*_RD_ for estimating the model parameters in the presence of six competing choices for the vector of nominal values. For both endpoints, *ξ*_RD_ performs the best across the six parameter sets. It also clearly outperforms the ad-hoc empirical designs *ξ*_*o*1_ and *ξ*_*o*2_. The uniform designs do not perform well despite their popularity; both graphs also suggest that more concentration points in the uniform designs do not necessarily result in better performance.

Figures 5 and 6 are plots of the c-efficiencies of the various designs across different sets of nominal values for the model parameters. Figure 5 shows the empirical design O1 is competitive with design *ξ*_RDc10_ but O2 outperforms *ξ*_RDc20_; however, Fig. 6 shows *ξ*_RDc25_ and *ξ*_RDc50_ clearly outperform all other designs, but their efficiencies are smaller than those observed for *ξ*_RDc10_ and *ξ*_RDc20_ in Figure 5. All three of the uniform designs consistently underperform for estimating the four lethal concentrations. For some set of nominal values, the difference in c-efficiencies between the robust design and one of the uniform designs can be as large as 39%, i.e., 62% versus 23% (for estimating LC_20_ ) with a similar magnitude difference for estimating LC_10_ as well (82% versus 41%). All the uniform designs *U*_4_, *U*_8_, and *U*_11_ perform poorly and uniformly worse than the robust designs for estimating all model parameters or the four lethal concentrations. Among them, they have mixed performances with no clear indication which one of the three is the best. The main practical implication is that intuitively appealing designs, such as uniform designs, can be very inefficient in that they require more time and labor to administer more doses yet can produce less reliable and less accurate estimates.

## Discussion

Optimal design theory was used to construct efficient designs for making statistical inference when a statistical model and a specific goal in the experiment were assumed. The optimal design provides the most accurate inference for a fixed amount of resources. However, the theory requires a known model and a clearly specified goal, neither of which is likely applicable in developmental toxicity studies. This is because frequently experiments are performed in an ad-hoc manner without a statistical model in mind. It is therefore useful to modify an optimal design which is robust to various model assumptions, different objectives, and different goals.

In this sea urchin developmental toxicity study, concentration–response relationships were established for trimethoprim and two developmental outcomes, namely aboral radialization and embryonic developmental arrest/death. The relationship was a sigmoidal curve with inhomogeneous variances on the responses and it was found that a Beta model provided a good fit to the data with concentration (dose) as the only independent variable. Using an algorithm developed by the coauthors [WKW and SWH], it was found D and LC-optimal designs were robust to different sets of nominal parameters for the Beta regression model. Statistical inference was sought for two endpoints in the sea urchin study: (i) estimating the model parameters and (ii) estimating various lethal concentrations. The optimal designs found from the algorithm for the Beta model were then compared to the ad-hoc empirical designs implemented for the sea urchin study and three uniform designs.

Although the Beta model provides a good fit to the data, the complexity of the model and the difficulty of finding an optimal design can deter the use of such a design in practice. To this end, a website was constructed to provide additional background information on design for such experiments and implement the algorithm so that a toxicologist can interactively use the site to generate different types of optimal designs.

The site address is https://elviscuihan.shinyapps.io/Dc_optimal_design/, and it allows a toxicologist to find the D-optimal design or the LC_p_-optimal design or a dual-objective optimal design for the two objectives after determination of the design parameters. The input parameters include the dose interval of interest, the value of *p*, up to six sets of possible values (Θ_1_, Θ_2_, Θ_3_, Θ_4_, Θ_5_, Θ_6_) for the nominal values of the Beta model and how long the algorithm is to be run, along with the grid size. For the latter two inputs, r and Grid, respectively, it is recommended to use the default values. If some sets of nominal values are deemed to be more likely than others, the user can assign an appropriate vector of probabilities (P1, P2, P3, P4, P5, P6) to reflect the plausibility of each set. If fewer than six sets of nominal values are available, the corresponding probabilities are assigned zeros. For finding a dual-objective optimal design, the user also needs to specify a value of W between 0 and 1 to indicate which of the two objectives are more important. Clearly, if the weight *W* used to balance between the two competing objectives is set to *W* = 0 or *W* = 1, the single-objective optimal design is obtained. After all inputs are provided, the user clicks on the “search optimal design!” prompt and it should take several seconds for the algorithm to find the desired optimal design.

In conclusion, some aspects of optimal design theory were used to construct optimal designs in a developmental toxicology study. Details on the theory of constructing and confirming optimality of a model-based design are omitted, but they can be found in Holland-Letz and Kopp-Schneider (2015), who described them nicely for a toxicology audience. Additional fundamentals of optimal design and technical details are also available in several design monographs, such as, Fedorov (1972), Silvey (1980), and Berger and Wong (2009). Examples of use of optimal design ideas in various fields are illustrated in many real applications in Berger and Wong (2005).

It is concluded that an optimal design is derived mathematically under a set of restrictive assumptions and so the optimal design may not meet all the practical requirements related to deriving a dose–response relationship. A guiding principle is that the implemented design should be modified to meet practical needs to the extent possible, and not deviate too much from the optimum to avoid a marked reduction in statistical efficiency. Some of the assumptions required for the theory may not be tenable in a laboratory experiment, but this should not totally exclude consideration of incorporating optimal design ideas in toxicology studies. For instance, model assumptions can be questionable, but data from the design can help validate the appropriateness of the assumed model by assessing the adequacy of the model fit. In addition, there are statistical tests to detect problems with specific model assumptions or overall adequacy of the performance of a model using an array of residual diagnostic tools (Cook and Weisberg 1982). The regression diagnostic results can then frequently provide a valuable guide to develop a more plausible model for the next experiment. The overarching problem is that there is a need to improve study design in toxicology experiments and incorporating some optimal design ideas at the design stage is a reasonable step in the right direction.

Therefore, a question that derives from this study is what does this mean for human developmental toxicity of trimethoprim? The answer is that trimethoprim is a developmental toxicant in sea urchins at the doses tested irrespective of whether the data are modeled or not. Further studies are required to determine the molecular basis of this toxicity in sea urchins, and then, a subsequent step would be to assess if this molecular pathway relates to mammalian (specifically human) development. These steps may be facilitated by having an accurate mathematical model of phenotypic results as well as the molecular events.
